# Posttraumatic stress in intensive care unit survivors – a prospective study

**DOI:** 10.1080/21642850.2014.943760

**Published:** 2014-08-20

**Authors:** Mette Ratzer, Ole Brink, Linda Knudsen, Ask Elklit

**Affiliations:** ^a^ Pedagogic Psychological Counseling, Aarhus Municipality, Aarhus, Denmark; ^b^ Trauma Center, Aarhus University Hospital, Aarhus, Denmark; ^c^ Department of Psychology, National Centre for Psychotraumatology, University of Southern Denmark, Odense, Denmark; ^d^ School of Psychology, University of Ulster Coleraine, Northern Ireland

**Keywords:** posttraumatic stress, intensive care, trauma, ICU survivors

## Abstract

*Aims*: This study aimed to estimate the prevalence of severe Posttraumatic Stress Disorder (PTSD) symptoms and to identify factors associated with PTSD in survivors of intensive care unit (ICU) treatment following traumatic injury. *Methods*: Fifty-two patients who were admitted to an ICU through the emergency ward following traumatic injury were prospectively followed. Information on injury severity and ICU treatment were obtained through medical records. Demographic information and measures of acute stress symptoms, experienced social support, coping style, sense of coherence (SOC) and locus of control were assessed within one-month post-accident (T1). At the six months follow-up (T2), PTSD was assessed with the Harvard Trauma Questionnaire (HTQ). *Results*: In the six months follow-up, 10 respondents (19.2%) had HTQ total scores reaching a level suggestive of PTSD (*N* = 52), and 11 respondents (21%) had symptom levels indicating subclinical PTSD. Female, five illness factors: coma time, mechanical ventilation, sedation, benzodiazepine, pain relieving medication, and four psychological factors: symptoms of acute stress (T1), fear of death and/or feeling completely helpless and powerless in relation to the accident and/or ICU (T1), SOC (T1) and more external locus of control (T1) correlated significantly with PTSD symptoms at T2. In the linear regression analysis, female, length of sedation, dissociation (T1), hypervigilance (T1), and external locus of control predicted 58% of the variation of PTSD. *Conclusions*: High levels of PTSD symptoms occurred in 19.2% of respondents in six months following traumatic injury requiring ICU admission. Screening for the variables gender, length of sedation, dissociation, hypervigilance, and locus of control after ICU admission following traumatic injuries may help to predict who will develop PTSD.

## 1. Background

Posttraumatic stress disorder (PTSD) is the development of a characteristic pattern of psychological and behavioral symptoms following exposure to extreme stress. Events that can trigger PTSD involve experiencing a serious threat to one's own physical integrity that is experienced with intense fear, horror, or helplessness. Diagnostic criteria for PTSD include exposure to one or more traumatic events and symptoms from each of three symptom clusters: intrusive recollections, avoidance/emotional numbing, and hyperarousal (APA, 2000).

Admission to an intensive care unit (ICU) exposes the patient to serious stressors, such as respiratory distress, being in pain, having tubes in nose and/or mouth, loss of control, sleep deprivation, physical restraint, and not being able to communicate (Biancofiore et al., 2005; Novaes et al., 1999; Skalski, DiGerolamo, & Gigliotti, 2006). Furthermore, delusions and hallucinations, which can be very frightening and persecutory in nature, are often associated with ICU treatment (Jones, Griffiths, Humphris, & Skirrow, 2001; Roberts, 2004; Roberts, Rickard, Rajbhandari, & Reynolds, 2006).

Reviews of the prevalence of posttraumatic stress reactions post-ICU treatment estimate that survivors of general ICU hospitalization have a considerable prevalence of PTSD symptoms (Davydow, Gifford, Desaim, Needham, & Bienvenu, 2008). Furthermore, prior studies have revealed that survivors of traumatic injuries are at risk of developing PTSD (O'Donnell, Creamer, Bryant, Schnyder, & Shalev, 2003; Zatzick et al., 2007). Both studies support the theory that admission to an ICU can be an independent stressor for the development of PTSD (O'Donnell et al., 2010; Zatzick et al., 2007). Davydow et al. (2009) have studied PTSD related to ICU treatment after trauma in a large sample and found that 25% had symptoms suggestive of PTSD.

### 1.1. Risk factors for PTSD in ICU populations

Previous studies have found that certain demographic, treatment, and psychological factors are related to the development of post-ICU PTSD symptoms. Among the demographic factors are younger age, female, educational level, and prior psychiatric history generally regarded as PTSD risk factors (Brewin, Andrews, & Valentine, 2000) and have been reported as risk factors in some ICU studies.

Treatment with benzodiazepines (Girard et al., 2007; Sackey, Martling, Carlswärd, Sundin, & Radell, 2008; Samuelson, Lundberg, & Fridlund, 2007), treatment with stress hormones (Schelling et al., 1999, 2001, 2004; Weis et al., 2006), and sedation practice (Kress et al., 2003; Weinert & Sprenkle, 2008) are some of the treatment factors possibly related to the development of post-ICU PTSD. According to a recent review (Ratzer, Romano, & Elklit, 2014), benzodiazepines and sedation practices are considered risk factors for the development of PTSD, while the administration of stress hormones seem to buffer against later PTSD symptoms. Furthermore, length of (LO) stay in ICU (Davydow et al., 2009; Hauer et al., 2009; Kapfhammer, Rothenhäusler, Krauseneck, Stoll, & Schelling, 2004; Rattray, Johnston, & Wildsmith 2005; Schelling et al., 1999; Stoll et al., 2000; Weis et al., 2006) and LO mechanical ventilation (MV, Cuthbertson, Hull, Strachan, & Scott, 2004; Davydow et al., 2009) have been confirmed predictors in a number of studies. Severity of illness has consistently been found *not* to be a significant predictor (Davydow, Gifford, Desaim, Needham, & Bienvenu, 2008). However, prior studies of post-ICU PTSD have found inconsistent results, regarding many of the potential risk factors (Davydow, Gifford, Desaim, Needham, & Bienvenu, 2008).

Psychological factors are not well studied in research focusing on post-ICU PTSD (Davydow et al., 2008). Several psychological factors have although evidenced with adaptation to traumatic stressors (Gibbs, 1989). Therefore, this study included five measures of (a) acute stress disorder (ASD), (b) coping styles, (c) experience of social support, (d) sense of coherence (SOC), and (e) locus of control as possible predictors for trauma level after six months.

*ASD* was introduced in the Diagnostic and Statistical Manual of Mental Disorders (American Psychiatric Association, 1994) with the purpose of (1) recognizing posttraumatic stress occurring *within the first month* after a traumatic event and (2) identifying the victims most at risk of developing PTSD (Harvey & Bryant, 2002). The primary differences between ASD and PTSD are the time criteria and the focus on *dissociation* symptoms (Bryant, 2007). Dissociation may be understood as a variety of responses observed in the course of a traumatic experience which causes disruption in the usually integrated functions of consciousness, perception of the environment, memory, or identity (American Psychiatric Association, 1994; Bryant, 2007). Examples of dissociative symptoms are a sense of numbing or detachment, reduced awareness of one's surroundings, derealization, depersonalization, or dissociative amnesia (American Psychiatric Association, 1994; Bryant, 2007). Dissociative responses occurring at the time of trauma (“peritraumatic dissociation”) are previously established as the largest known risk factor for the development of PTSD (Ozer, Best, Lipsey, & Weiss, 2003). In contrast to that, some researchers have found the ASD diagnosis to be of limited use in predicting PTSD (Elklit & Christiansen, 2010; Harvey & Bryant, 2002).

Different *coping styles* have been previously associated with the development of PTSD. Problem-oriented coping shortly after the accident predicted PTSD at the one-year follow-up in the study by Schnyder, Moergeli, Klaghofer, and Buddeberg (2001). Bryant, Marosszeky, Crooks, Baguley, and Gurka (2000) found that avoidant coping style and behavioral coping style were significant predictors of PTSD severity following severe traumatic brain injury (TBI). In a study by Nielsen (2003), emotional coping predicted PTSD in persons with spinal cord lesions.

Only a few of the reviewed studies have included examination of the relation between post-ICU PTSD and experienced *social support* (Deja et al., 2006, Kapfhammer et al., 2004). In general, a perception of lack of social support is found to be a strong predictor of PTSD across different study populations (Brewin et al., 2000). This was confirmed in the study by Deja et al. (2006), but not in the study by Kapfhammer et al. (2004) or in a recent study on accident victims (Baranyi et al., 2010). In a study comparable to the present study, regarding baseline and follow-up time points, Dougall, Ursano, Posluszny, Fullerton, and Baum (2001) found that the level of social support at two to three weeks post-motor vehicle accident (MVA) was lower in the six months PTSD group than in the non-PTSD group. *SOC* (Antonovsky, 1987) is defined as a global orientation that expresses the extent to which one has a pervasive, enduring (although dynamic) feeling of confidence (Antonovsky, 1987). Research has supported the conception of SOC as resilience to stress disposition as high SOC has been associated with lower risk of pathological stress reactions (Fromberger et al., 1999; Fuglsang, Moergeli, Hepp-Beg, & Schnyder, 2002; Hepp et al., 2008). A lower SOC has previously been associated with higher levels of psychological distress following trauma (Fromberger et al., 1999; Fuglsang et al., 2002; Hepp, Moergeli, Büchi, Wittmann, & Schnyder, 2005). Locus of control is defined as the extent to which the individual judges outcomes to be contingent on his or her behavior (Rotter, 1966). External locus of control has previously been associated with poorer psychological adjustments following traumatic experiences (Chung, Preveza, Papandreou, & Prevezas, 2007; Kushner, Riggs, Foa, & Miller, 1992).

Hypotheses for the study were:

1. There is a positive association between measures of ASD < one month after the admission to ICU and PTSD symptoms six months post-accident.
2. The study will be explorative pertaining to the various kinds of medicaments and invasive procedures generally expecting them to be positively associated with symptoms of PTSD.
3. There is a negative association between LO coma and sedation and PTSD symptoms.
4. We expect rational and detached coping, positive social support, and internal locus of control to be associated with lower levels of PTSD.

## 2. Methods

### 2.1. Design and settings

The study was a Danish prospective cohort study designed to assess the long-term psychological sequelae of ICU treatment at the University Hospital of Århus following traumatic injury. The study adheres to the general demands of ethical research of the Helsinki Declaration (World Medical Association, 2000). The study was approved by the regional ethical scientific committee, and the Danish Data Inspection Authority.

### 2.2. Patients population

**Figure 1.**
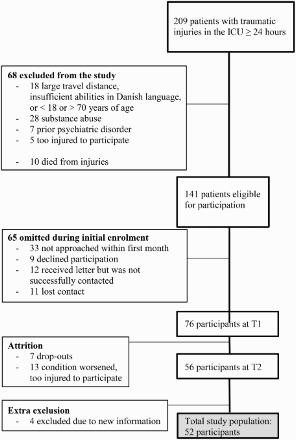
Flow chart of participant enrollment.

All qualifying patients had sustained traumatic injuries that required referral to ICU between January 2007 and December 2008. A participant flow chart from referral to the ICU to final study population is presented in Figure 1. Patients were included if they were between 18 and 70 years, stayed in ICU > 24 hours, and had sufficient abilities in Danish language and a clinical condition enabling participation in an extensive clinical interview within one month of the accident. Exclusion criteria were: any serious somatic illness or treatment for any mental disorder immediately prior to the accident, and/or showing any marked clinical signs or symptoms of mental disorders obviously unrelated to the accident, substance abuse, or referral due to suicide attempt.

During the screening period, 209 patients fulfilled the inclusion criteria of a stay in the ICU for at least 24 hours and were enrolled in the study. Of the 209 enrolled patients, 18 patients were excluded due to age, insufficient Danish language proficiency or living too far away. Another 28 were excluded for substance abuse and 7 for prior psychiatric disorder. Some of the patients had both substance abuse and psychiatric disorder, but figure in only one of the categories. Ten died of their injuries. Five patients were too injured to participate.

One-hundred and forty-one patients were eligible for participation. Thirty-three patients were not approached within the first month (missed). Twelve patients were never successfully contacted even though they received a letter. Eleven patients were not tested because we lost contact with them. Nine patients did not want to participate. Twenty-seven patients, who were too injured to participate at T1, were included at T2, but do not figure in this article because of the missing data from T1.

Of the 141 eligible patients, 76 patients participated in the < one-month interview and of those 56 also participated in the second interview (7 patients lost to follow-up between T1 and T2, and the conditions of 13 patients worsened between T1 and T2 and were thereby too injured to participate at T2). Furthermore four patients were excluded because information came forth at T1 that excluded the respondents. The study population of this article is the 52 patients responding at both T1 and T2 and at T2 responding on the Harvard Trauma Questionnaire (HTQ).

### 2.3. Procedure

Information regarding demographic, injury-related, and treatment factors were obtained from medical records and trauma registries at the hospital. In-person assessments of psychological variables were conducted within one-month post-accident (T1) and at six months post-accident (T2) by a clinical psychologist or trained psychology student.

### 2.4. Instruments

Injury severity was measured by the Injury Severity Score (ISS, Baker, O'Neill, Haddon, & Long, 1974; Copes et al., 1988) and the Glasgow Coma Scale (GCS, Sternbach, 2000; Teasdale & Jennett, 1974). The *ISS* is an anatomical scoring system that provides an overall score for patients with multiple injuries and is based upon the Abbreviated Injury Scale (AIS). The AIS describes the severity of injury to one body region: (1) minor, (2) moderate, (3) serious, (4) severe, (5) critical, and (6) un-survivable. To calculate an ISS for an injured person, the body is divided into six regions. These body regions are: head and neck, including cervical spine, face, including the facial skeleton, nose, mouth, eyes and ears, thorax, thoracic spine and diaphragm, abdomen, abdominal organs and lumbar spine, and extremities including pelvic skeleton, external soft tissue injury. The three most severely injured body regions have their scores squared and added together to produce the ISS score. The ISS takes scores from 0 to 75 (i.e. AIS scores of 5 for each category). If any of the three scores is a 6, the score is automatically set at 75 (Baker et al., 1974; Copes et al., 1988). The *GCS* is used to assess a person's state of consciousness. The person is scored in three categories; eyes, motor response, and verbal response. The maximal score is 15, meaning a fully awake person and the minimum score is 3, for at person deeply unconscious (Sternbach, 2000; Teasdale & Jennett, 1974). The GCS is used in the categorical division of severity of head injury by the GCS score of mild (13–15), moderate (9–12), and severe (8 or less) (Rimel, Giordani, Barth, & Jane, 1982; Sternbach, 2000).

Scales for measuring the psychological symptoms of posttraumatic stress were the Acute Stress Disorder Scale (ASDS, Bryant, Moulds, & Guthrie, 2000) at T1 and the HTQ (Mollica et al., 1992) at T2. The ASDS is a self-report measure based on the dissociative, re-experiencing, avoidance, and arousal symptoms listed in the *DSM-IV*. The scale possesses fine psychometric qualities (Bryant, Marosszeky, Crooks, & Gurka, 2000). The ASDS consists of 19 questions relating to each of the ASD symptom clusters. Using a 5-point Likert Scale ranging from 1 (*not at all*) to 5 (*very much*) respondents indicate the intensity of each symptom. The possible range for total score is 19–95, and the possible score range on the subscales is 5–25 (dissociation), 4–20 (re-experiencing), 4–20 (avoidance), and 6–30 (arousal). Individual item scores above 3 indicate symptom presence. Cronbach's alpha values for the present study were 0.84 for the total scale, 0.71 for re-experiencing, 0.55 for avoidance, 0.75 for dissociation, and 0.69 for hypervigilance.

The HTQ estimated the occurrence of PTSD at T2. It consists of 32 items scored on a 4-point Likert Scale. The scale measures the intensity of the three PTSD core symptom clusters (intrusion, avoidance, and arousal). The subscales are scored separately. Only scale items ≥ 3 were counted toward a PTSD diagnosis. Mollica et al. (1992) found the original scale reliable and valid. Self-report measure of PTSD had 88% concordance with interview-based estimates of PTSD ( Mollica et al. 1992). The Cronbach's alpha value in the present study was 0.92. (total scale) and for the subscales 0.77 (intrusion), 0.64 (avoidance), and 0.77 (arousal).

Coping style was measured by the Coping Style Questionnaire (CSQ; Roger, Jarvis, & Najarian, 1993). The original version consists of 60 questions, measured on a 4-point Likert Scale ranging from “never” (1) to “always” (4). The version of the CSQ used in this study consists of 37 questions and was developed in a factor analytic study of the original 60-item version of the scale (Elklit, 1996).

The Crisis Support Scale (CSS; Joseph, Andrews, Williams, & Yule, 1992) measured social support at T1. The seven questions (7-point Likert Scale) relate to perceived available support, practical help, and emotional support, contact with other bereaved, ability to express thoughts and emotions related to the loss, the degree to which one feels let down, and general satisfaction with social support. The scale has good psychometric qualities (Elklit, Pedersen, & Jind, 2001). Cronbach's alpha for the present study was 0.57, which is lower than that for the study by Elklit et al. (2001).

The Sense of Coherence Scale (SOC; Antonovsky, 1987) is a self-report measure of the degree to which an individual views his or her world as being comprehensible (eleven items, e.g. “When you talk to people, do you have the feeling that they don't understand you?”), manageable (ten items, e.g. “Has it happened that people whom you counted on disappointed you?”), and meaningful (eight items, e.g. “Do you have the feeling that you don't really care about what goes on around you?”). Respondents indicate the extent to which they agree or disagree with the items, and responses to all items are made by means of 7-point Likert Scales. Higher scores indicate a stronger SOC. The 29-item version was used in this study. Test properties such as test–retest reliability and internal consistency of the SOC are excellent (Antonovsky, 1993). Cronbach's alpha values for the present study were 0.77 (manageability), 0.85 (meaning), 0.71 (comprehension), and 0.87 (total scale).

Locus of control was measured by a visual analog scale, with one pole expressing the phrase: “I am normally in control of the things that happen to me” and the other pole expressing the phrase “I don't feel that I have any control of the direction that my life is taking”.

### 2.5. Statistical analysis

Proportions are reported as raw numbers and percentages. Means are reported with standard deviations and ranges. Correlations were estimated with Pearson's correlation coefficient. ANOVA analysis was used to investigate complex associations between variables. Multiple linear regression analysis was used to assess the effect of predictor variables on the HTQ total scores.

## 3. Results

The following presentation of the results starts with a description of the demographic and clinical characteristics of the study population (Table 1) and a comparison of respondents versus all eligible patients (Table 2). Table 3 presents the frequencies of ICU treatment characteristics. These are followed by a description of the prevalence of symptoms indicative of PTSD. Analysis of the associations between PTSD symptomatology and potential risk factors are presented (Table 4), and finally a hierarchical regression analysis with the HTQ score as the dependent variable is made (Table 5).

**Table 1.** Characteristics of respondents in the six months follow-up (*N* = 52).

	Mean (SD, range)/yes
Age (years)	40.4 (15.9, 18–69)
Gender (M/F, %)	57.7/42.3
*Marital status*	
Living with partner (%)	51.9
Living alone (%)	48.1
Having one or more children (%)	55.8
Mean number of children	1.4 (1.5, 0–5)
Number of previous traumas	2.2 (1.6, 0–8)
One or more previous trauma (%)	88.2 (*N* = 51)
ICU LOS (days)	5.4 (5.5, 1–28)
ISS score	16.5 (8.7, 4–43)
GCS score at arrival	11.1 (5.3, 3–15)
Head trauma (%)	52.9 (*N* = 51)
Mean head AIS	1.4 (1.6, 0–5)
MV (%)	40.4
Mean LO MV	1.7 (4.1, 0–22)
Fear of death (T1) (%)	18.4 (*N* = 49)
Feeling helpless and powerless (T1) (%)	60.8 (*N* = 51)
One or both of the above feelings = PTSD A2 criterion (%)	61.2% (*N* = 49)
Injury mechanism	(*N* = 43)
MVA (%)	72.1
Bicycle (%)	9.3
Horse (%)	2.3
Fall (%)	7.0
Other (%)	2.3
Missing (%)	7.0

Note: ICU = Intensive Care Unit, LOS = Length of Stay, ISS = Injury Severity Scale, GCS = GlasgowComa Scale, LO = Length of, and MV = Mechanical Ventilation.

**Table 2.** Comparison of responders and all eligible patients.

	Respondents in the six months follow-up	All eligible patients
	*N* = 52 Mean (SD, range)	*N* = 141 Mean (SD, range)
Age (years)	40.4 (15.9; 18–69)	40.0 (16.0; 18–70) (*N* = 141)
Gender (M/F, %)	57.7/42.3%	71.6/28.4% (*N* = 141)
ICU LOS (days)	5.4 (5.5; 1–28)	8.9 (9.8; 1–57.5) *N* = 109
ISS score	16.5 (8.7; 4–43)	18.9 (9.6; 4–50) *N* = 115
GCS score at arrival	11.1 (5.3; 3–15)	10.1 (5.6; 3–15) *n* = 109
MV/resp. treatment (%)	40.4%	54.2% (*N* = 107)
Mean LO MV	1.7 (4.1; 0–22)	5.3 days (8.7; 0–48.25) (*N* = 106)

Note: ICU = Intensive Care Unit, LOS = Length of Stay, ISS = Injury Severity Scale, GCS = Glasgow Coma Scale, MV = Mechanical Ventilation, and LO = Length of.

**Table 3.** Description of ICU treatment frequencies for respondents in the six-month follow-up.

ICU treatment	Yes
*Sedation*	38.5%
Midazolam (benzodiazepine)	19.2%
Propofol	38.5%
*Stress hormones*	
Sympathomimetics pere op.	31.4%
Parasympathomimetics pere op.	5.9%
Sympathomimetics int.	23.1%
Parasympath. Int.	0%
Hydrocortisone (steroid)	11.5%
*LO coma*	
Unconscious (spontaneously)^a^	28.8%
*Mean LO unconsciousness*	
Hours	0.16 (0.6, 0–4)
Days	0.61 (2.3, 0–13)
Unconscious (by sedation)	38.5%
Mean length of sedation (days)	0.9 (2.4, 0–14.8)
Awakened during sedation	9.6%
Tracheotomy	5.8%
Fentanyl	48.1%
Remifentanyl	11.5%
Psychopharmacological drugs	11.5%
Intensive delirium	0%

Note: ICU = Intensive Care Unit, LO = Length of.

^a^Some patients figure in both categories of unconsciousness.

**Table 4.** Pearson Correlations and ANOVA *F*-ratios between HTQ total and demographic, illness, treatment and psychological characteristics.

	*r*/*F*	*P*	*N*
Age	*r* = 0.05	.72	52
*Gender*	*F* = 8.82	.005	52
Marital status	*r* = 0.09	.35	52
Number of children	*r* = 0.05	.64	52
Number of previous	*r* = 0.22	.12	51
*Traumas*			
Pre-injury use of medication	*F* = 1.46	.23	52
Time since accident	*r* = 0.21	.15	50
ISS	*r* = −0.06	.65	51
AIS head	*r* = 0.11	.46	51
*GCS arrival*	*r* = −0.31	.03	52
LOS ICU	*r* = 0.17	.23	52
*LO MV*	*r* = 0.31	.03	52
*LO Unconsciousness*			
Spontaneously			
(1) Hours	*r* = −0.11	.42	52
(2) Days	*r* = 0.18	.20	52
*LO sedation*	*r* = 0.39	.005	52
Awakened during	*r* = 0.82	.37	52
*Sedation*			
*LO Midazolam sedation*	*r* = 0.35	.01	52
LO Propofol sedation	*r* = 0.26	.06	52
*LO Fentanyl int.*	*r* = 0.37	.01	52
*LO Remifenta. Int.*	*r* = 0.29	.04	52
Psychopharmacological drugs	*F* = 2.95	.09	52
Tracheotomy	*F* = 3.57	.06	52
*Fear of death* (T1)	*F* = 7.63	.01	49
*Feeling completely helpless and powerless* (T1)	*F* = 7.55	.01	51
*ASDS total* (*T1*)	*r* = 0.67	.00	50
*Intrusion*	*r* = 0.36	.01	50
*Hypervigilance*	*r* = 0.56	.00	50
*Dissociation*	*r* = 0.67	.00	51
*Avoidance*	*r* = 0.38	.02	50
CSS total (T1)	*r* = 0.03	.86	49
*SOC* (T1)			
*Comprehension*	*R* = −0.50	.001	43
*Manageability*	*r* = −0.40	.01	44
*Meaning*	*r* = −0.18	.21	49
*Locus of control* (T1)	*r* = 0.39	.005	50
*CSQ* (T1)			
Rational coping (T1)	*r* = −.05	.72	48
Emotional coping (T1)	*r* = 0.17	.25	49
Detached coping (T1)	*r* = −0.07	.64	49
Avoidant coping (T1)	*r* = 0.21	.16	47

Note: HTQ = Harvard Trauma Questionnaire, ISS = Injury Severity Scale, AIS = Abbreviated Injury Score, GCS = Glasgow Coma Scale, LOS = Length of Stay, ICU = Intensive Care Unit, LO = Length of, MV = Mechanical Ventilation, ASDS = Acute Stress Disorder Scale, CSS = Crisis Support Scale, SOC = Sense of Coherence, and CSQ = Coping Styles Questionnaire.

**Table 5.** Hierarchical linear regression analysis of the examined factors contributions in the prediction of the variance in the HTQ total score at the six months follow-up.

Step	Variable	*β*	*P*	*F*	df	Adj. *R*²	*R*²ch	*P*
1	Gender	.37	.01	7.63	1.49	.12	.14	.008
2	Gender	.33	.02	9.09	2.49	.25	.28	.000
	LO sedation	.38	.005					
3	Gender	.22	.05	15.89	3.49	.48	.51	.000
	LO sedation	.24	.05					
	ASDS dissociation	.52	.0005					
4	Gender	.19	.06	16.00	4.49	.55	.59	.000
	LO sedation	.24	.02					
	ASDS dissociation	.37	.005					
	ASDS hypervigiliance	.32	.005					
5	Gender	.19	.05	14.50	5.49	.58	.62	.000
	LO sedation	.24	.02					
	ASDS dissociation	.33	.005					
	ASDS hypervigiliance	.29	.01					
	VAS locus of control	.20	.05					

Note: HTQ = Harvard Trauma Questionnaire, LO = Length of, ASDS = Acute Stress Disorder Scale, and VAS = Visual Analogue Scale.

### 3.1. Demographic and clinical characteristic/descriptive data

The baseline characteristics of the respondents at the six months follow-up are presented in Table 1. Of the head trauma patients, 6 patients had a GCS score between 13 and 14 (mild TBI), 4 patients had a GCS score between 9 and 12 (moderate TBI), and 15 patients had a GCS score between 3 and 8 (severe TBI).

Of the total study population, 27 (51.9%) were admitted to a Neuro Intensive Unit and 22 (42.3%) were admitted to an Intensive Therapy Unit, which is a multidisciplinary ICU. Three patients (5.8%) were treated in both units. The respondents tended to be less seriously injured and were more likely to be female compared to the entire eligible sample (Table 2). The ICU treatment characteristics are given in Table 3.

### 3.2. Prevalence of PTSD symptoms

The respondents were interviewed within one-month post-accident (mean 25.9 days, SD 9.3, range 1–60) and again at the six months follow-up (mean 6.2 months, SD 0.5, range 5–8). At the six months follow-up, 10 respondents (19.2%) had HTQ total scores reaching a level suggestive of PTSD (*N* = 52). In addition, 11 respondents (21%) met criteria for two symptom clusters, which correspond to a subclinical PTSD symptom level.

### 3.3. Factors associated with symptoms of posttraumatic stress

#### 3.3.1. Correlation and analysis of variance

Pearson correlations or ANOVA analyses were computed between HTQ total scores and all other variables to identify factors associated with symptoms of posttraumatic stress six months post-ICU admission following traumatic injuries. The correlations and ANOVA analyses between HTQ total and demographic, illness, treatment, and psychological variables are given in Table 4. One-way ANOVA analysis showed that female respondents had significantly higher HTQ total score six months post-ICU admission (Table 4).

The HTQ total score correlated negatively with the GCS at arrival and positively with LO MV. An ANOVA analysis of the variance between the group of respondents with a GCS score equal to or below 8 indicating severe TBI and the respondents with a GCS score of 9 and above showed a significant variance in the HTQ total score between the groups *F*(1,51) = 5.71, *p* = .02). The HTQ total score did not correlate significantly with illness severity (ISS), LO stay in ICU, or LO unconsciousness.

There were significant positive correlations between the HTQ total score and the treatment variables LO sedation and LO Midazolam sedation (Table 4). Furthermore, LO treatment with the pain relievers Fentanyl and Remifentanyl correlated positively with the HTQ total score. LO MV and the significant treatment variables were all highly intercorrelated with the exception of the LO Remifentanyl intravenous.

One-way ANOVA analysis showed correlations between HTQ total and measures of the A2 criteria within one-month post-accident: fear of death and feeling completely helpless and powerless. Each of the two variables reflects fulfilling of the PTSD A2 criteria. At T1, 31 respondents (60.8%, *N* = 51) fulfilled either or both measures of the A2 criterion. The association between A2 (T1) and HTQ total was significant (*F*(1,49) = 6.46, *p* = .02).

There were significant positive correlations between the HTQ total score and the ASDS total score and the four ASDS subscales (Table 4). At T1, seven respondents (14%) had ASDS scores reaching a level suggestive of ASD within one-month post-injury (*N* = 50). The ASDS mean total score was 34.0 (SD 10.5 range 19–62), the mean dissociation score 11.2 (SD 4.7, range 5–23), the mean avoidance score 5.4 (SD 2.1, range 4–12), the mean hypervigilance score was 11.5 (SD 4.2, range 6–23), and the mean intrusion score was 6.2 (SD 2.8, range 4–15).

Regarding the categorical relation between ASD (T1) and PTSD (T2) (*N* = 50): of the seven respondents with symptom level indicating ASD only one had HTQ total scores indicating PTSD and three had a level indicating subclinical PTSD. The SOC manageability and comprehension scales correlated inversely with the HTQ total score, but the SOC meaning did not (Table 4). The CSS scores (mean total = 38.7) indicated that most respondents generally experienced good support from their social network and were able to express their feelings.

At T1, the mean locus of control was 3.5 (SD 2.4, range 0–10). The higher the score the more external locus of control the respondent is experiencing. There was a significant correlation between the HTQ total (T2) and locus of control (T1) (*r* = 0.39, *p* = .005, *N* = 51).

#### 3.3.2. Regression analysis

A linear regression analysis was made with the HTQ total scores (T2) as the dependent variable and is given in Table 5. Factors that correlated significantly with the HTQ total score were entered into a series of regression models. Factors that did not correlate significantly with the HTQ scores, but were assumed important based on previous studies were also entered into the regression models. The variables were entered systematically in order of demography, illness, treatment, and psychological predictors. In the final model gender, LO sedation, ASDS dissociation, ASDS hypervigilance, and locus of control (T1) predicted 58% of the variation in the HTQ total score (T2).

## 4. Discussion

This study found that 19.2% of the survivors of ICU treatment following traumatic injuries prevalence symptoms were at risk of PTSD and that, additionally, 21% had symptoms indicating subclinical PTSD. The prevalence is consistent with the prevalence found in other studies of ICU survivors (Davydow et al., 2008). The finding that females are at greater risk of developing PTSD is in line with the existing literature (Brewin et al., 2000).

The *first* hypothesis that the ASD at T1 would be positively associated with PTSD at the six months follow-up was confirmed. The results concerning the categorical relations between ASD and PTSD do not support a full diagnosis of ASD as a satisfying predictor of later PTSD (Elklit & Christiansen, 2010; Harvey & Bryant, 2002). However, at the correlation level, the hypothesis was confirmed. Especially dissociation symptoms and the symptoms of hypervigilance were strong predictors of PTSD symptomatology.

The strong predictive effect of dissociation is in line with a large meta-analysis which found peritraumatic dissociation to be the strongest predictor of subsequent PTSD (Ozer et al., 2003). Several other studies have similarly found a strong relationship between early dissociation and later PTSD in injured trauma survivors (Shalev, Peri, Canetti, & Schreiber, 1996), in MVA survivors (Ehring, Ehlers, Cleare & Glucksman, 2008) and in a study of PTSD following mild TBI (Bryant & Harvey, 1998). The strong predictive values of dissociation and hypervigilance found in this study could be explained by considering the special population of accident/ICU survivors. One could argue that the strong association between PTSD and the ASD subscales dissociation and hypervigilance reflects that these are more bodily stress responses, which are less dependent on memory for the traumatic event.

Findings regarding the association between dissociation and PTSD have been criticized for conceptual problems because peritraumatic responses such as dissociation might themselves be aspects of the outcome we wish to explain. It is argued that dissociations, negative appraisal, and PTSD are likely to be manifestation of the same psychological process or consequences of a common vulnerability (Breslau, 2009). Furthermore, symptoms like amnesia for the traumatic event reduced awareness and derealization which are included in the ASDS dissociation cluster could be due to physical circumstances (e.g. the injury). Likewise, restlessness, insomnia, irritability, arousal, and concentration difficulties, which are included in the ASDS hypervigilance cluster, are also explainable due to the physical circumstances in ICU/accident survivors.

This argumentation is in line with Creamer, O'Donnell, and Pattison, (2004) who have argued that ASD is likely to be overestimated in severely injured trauma survivors due to organic/physical symptoms being taken as symptoms of ASD in questionnaire-ascertained estimations. However, this does not change the fact that early hypervigilance and especially dissociation were strong predictors of later PTSD symptomatology. The lower intrusion and avoidance scores also seem reasonable in a sample of survivors where many were unconscious or sedated during some of the stressing periods and many were still hospitalized at T1. Unconsciousness and sedation might have offered some protection of intrusive memories. The lower avoidance levels could be explained by the respondents not having resumed “normal” daily activities/ still being hospitalized as avoidant behavior to some extent implies the individual's freedom of action.

According to the *second* hypothesis, it was expected that some injury severity factors and some ICU treatment factor would be associated with symptoms of PTSD. As expected, the ISS score was not a predictor of six months PTSD. Interestingly, the GCS score was inversely related to symptoms of PTSD. This result indicates that the severity of head injury is positively associated with symptoms of PTSD.

Based on the literature on TBI it was expected that severe head injury could be a protective factor while milder head injuries were assumed to be either risk factor of neutral in relation to PTSD (King, 2008). This hypothesis was not supported. Rather, this is supportive of the research by Bryant, Marosszeky, Crooks, et al., (2000), which found PTSD following severe head trauma. Because diagnostic data regarding TBI (e.g. head scans and/or posttraumatic amnesia (PTA) were not collected, is it not possible to draw any absolute conclusions regarding the relation between TBI and PTSD, but the results support that symptoms of PTSD can develop in spite of severe head injuries. In line with the argumentation on the issue of TBI, LO coma was also hypothesized (#3) to protect against PTSD. However, the results indicate that LO coma (either spontaneously or sedated) and TBI do not seem to protect against symptoms of PTSD. Since ICU admission itself can be regarded as a risk factor, it is difficult to determine whether the accident or the loss of control, or invasive medical procedures in ICU is the eliciting stressor. The fact that ICU treatment in itself can be a stressor for PTSD is one possible way of explaining the co-existence of TBI and PTSD.

Bryant (1996) has suggested two possible explanations of the co-existence of TBI and PTSD. First, it is suggested that memories and feelings related to the stressor can develop by means of post-rationalization or later construction of memories based on information from family members or health personnel. Second, it is suggested that the co-existence of TBI and PTSD can be explained by an implicit level of encoding of the traumatic event (Bryant, 1996). The significant correlation between lengths of treatment with the benzodiazepine Midazolam confirmed previous findings associating benzodiazepines with the development of PTSD (Girard et al., 2007; Samuelson et al., 2007).

LO treatment with intravenous synthetic opioids against pain (Fentanyl and Remifentanyl) correlated positively with symptoms of PTSD. This result does not support recent findings that the use of morphine during early resuscitation and trauma care was significantly associated with a lower risk of PTSD after injury in military personnel (Holbrook, Anderson, Sieber, Browner, & Hoyt 1999) and in trauma patients (Bryant et al., 2009).

It has been suggested that delirium could be an independent predictor of post-ICU PTSD (Davydow et al., 2008). None of the respondents in this study was registered as having had delirium during the ICU stay. However, research has shown that intensive delirium is likely to be underreported (Ely et al., 2004). The fact that some patients received psychopharmacological drugs could be an indication of patients with undiscovered delirium. Davydow et al. (2008) have pointed at benzodiazepine sedation as an indicator of delirium. They argue that delirium could be an intermediate mechanism between benzodiazepines and post-ICU PTSD, reasoning that the administration of benzodiazepines may reflect the clinician's management of patients' anxiety or agitation. In this study, LO benzodiazepine administration correlated significantly with the symptoms of PTSD. In a further analysis of the data it could be interesting to examine the relation between the injury and ICU treatment factors relation to ASD and especially the dissociation and hypervigilance clusters.

According to the final hypothesis, it was expected that some coping strategies, social support perceptions, and cognitive attributions would mediate the associations between trauma severity and PTSD symptoms. This hypothesis was only confirmed regarding some of the psychological factors. None of the coping strategies (CSQ) correlated with symptoms of PTSD. This result does not support the finding from other related studies in which one or more coping styles predicted PTSD severity (Bryant, Marosszeky, Crooks, et al., 2000; Hepp et al., 2008; Nielsen, 2003; Schnyder et al., 2001). The finding that social support did not correlate with PTSD symptoms does not confirm the association between PTSD symptoms and social support found in Dougall et al. (2001). Thus, the findings regarding social support reported by Kapfhammer et al. (2004) and Baranyi et al. (2010) are supported. SOC comprehension and manageability correlated inversely with the PTSD symptomatology. The finding that low SOC score is related to higher PTSD scores corresponds to the understanding of the SOC as an indicator of the individuals resistance to stress in accident victims (Fromberger et al., 1999; Fuglsang et al., 2002; Hepp et al., 2005). In a study by Schnyder et al. (2001), the SOC score correlated significantly with the PTSD total score, but was not significant in the regression analysis. Hepp et al. (2005) studying the same population divided the sample into low versus high symptom group and found that the low symptom group had higher SOC scores. The strong correlation between SOC and PTSD found in this study indicates that in studying risk factors for psychological outcome following traumatic injuries/ICU admission individual trait factors should also be considered. Locus of control was a significant predictor of PTSD, as the more external locus of control experienced, the higher the HTQ total score. Given the simple methodological level of the instrument measuring locus of control in this study, the importance of this variable should not be overestimated. Nevertheless, the results indicate that more external locus of control may have an influence on the development of PTSD symptoms after a serious accident. This finding support other findings that associate external locus of control with poorer psychological adjustments following accidents (Chung et al., 2007) and crime victims (Kushner et al., 1992).

With the five factors, gender, LO sedation, dissociation (T1), hypervigilance (T1), and locus of control (T1), 58% of the variance in the (T2) HTQ score could be predicted. These are factors that are possible to examine shortly after the accident and ICU admission. This suggests that assessing these factors would help to predict those patients who were at risk of developing PTSD. Increased control or assessment of these risk factors among ICU patients may aid in treating or preventing PTSD symptomatology post-ICU treatment. Furthermore, dissociative and hypervigilance symptoms must be attended to in psychological treatment of patients with PTSD symptoms post-ICU treatment.

A strength of this study is the consideration of both demographic-, injury severity-, medical-, and psychological variables. Although not all manifesting themselves in the regression analysis, variables from all four categories were significant in the correlations with six months PTSD symptomatology. Furthermore, the study is strengthened by the prospective longitudinal design.

However, there are several limitations in this study. The small sample size, the low response rate, and the one-sited design question the generalization to posttrauma ICU patients in general. Zatzick et al. (2007) revealed variation between the different hospitals in the prevalence of post-accidental PTSD. Another limitation is the exclusion of patients with prior psychiatric disorders and/or substance abuse, which potentially biased the sample in the direction of higher psychosocial resources. Since psychiatric history is a well-established risk factor for PTSD (Brewin et al., 2000), the prevalence rate is likely to be higher. The fact that responders and non-responders differed regarding gender distribution and injury severity implies further caution regarding generalizing the results. Furthermore, relying on questionnaire-ascertained measures of PTSD includes a risk of estimating a false positive PTSD diagnosis. In line with this limitation, only 61.2% of the respondents reported having felt fear of death and/or completely helpless and powerless in relation to the accident or ICU admission, and therefore the PTSD A2 criteria were probably not met by all respondents. The number of previous trauma did not correlate significantly with symptoms of PTSD, however as we did not ask for other recent traumatic experiences PTSD symptoms could be related to other traumatic experiences. For determination of TBI we used the GCS in combination with head injury. We did not have measures of PTA, whereas other studies have used the combination of GCS score and PTA length to determine the degree of TBI (Bryant, Marosszeky, Crooks, et al., 2000).

## 5. Conclusions

The findings of this study confirm that a considerable number (40%) of the survivors of ICU following traumatic injuries is at risk of developing full or subclinical PTSD. When one also considers the physical state of the non-responders with much higher level of injury, attention to and assessment of psychological trauma reactions immediately and for a follow-up period should be part of a standard ICU procedure.

## References

[CIT0001] American Psychiatric Association (1994). *Diagnostic and statistical manual of mental disorders*.

[CIT0002] American Psychiatric Association (2000). *Diagnostic and statistical manual of mental disorders – TR*.

[CIT0003] Antonovsky A. (1987). *Unraveling the mystery of health: How people manage stress and stay well*.

[CIT0004] Antonovsky A. (1993). The structure and properties of the sense of coherence scale. *Social Science & Medicine*.

[CIT0005] Baker S. P., O'Neill B., Haddon W., Long W. B. (1974). The injury severity score: A method for describing patients with multiple injuries and evaluating emergency care. *Journal of Trauma*.

[CIT0006] Baranyi A., Leithgöb O., Kreiner B., Tanzer K., Ehrlich G., Hofer H. P., Rothenhäusler H. B. (2010). Relationship between posttraumatic stress disorder, quality of life, social support, and affective and dissociative status in severely injured accident victims 12 months after trauma. *Psychosomatics*.

[CIT0007] Biancofiore G., Bindi M. L., Romanelli A. M., Urbani L., Mosca F., Filipponi F. (2005). Stress-inducing factors in ICUs: What liver transplant recipients experience and what caregivers perceive. *Liver Transplantation*.

[CIT0008] Breslau N. (2009). The epidemiology of trauma, PTSD, and other posttrauma disorders. *Trauma, Violence, & Abuse*.

[CIT0009] Brewin C. R., Andrews B., Valentine J. D. (2000). Meta-analysis of risk factors for posttraumatic stress disorder in trauma-exposed adults. *Journal of Consulting and Clinical Psychology*.

[CIT0010] Bryant R. A. (1996). Posttraumatic stress disorder, flashbacks and pseudomemories in closed head injury. *Journal of Traumatic Stress*.

[CIT0011] Bryant R. A. (2007). Does dissociation further our understanding of PTSD?. *Journal of Anxiety Disorders*.

[CIT0012] Bryant R. A., Creamer M., O'Donnell M., Silove D., Clark C. R., McFarlane A. C. (2009). Post-traumatic amnesia and the nature of post-traumatic stress disorder after mild traumatic brain injury. *Journal of the International Neuropsychological Society*.

[CIT0013] Bryant R. A., Harvey A. G. (1998). Relationship between acute stress disorder and posttraumatic stress disorder following mild traumatic brain injury. *American Journal of Psychiatry*.

[CIT0014] Bryant R. A., Marosszeky J. E., Crooks J., Baguley I., Gurka J. (2000). Coping style and post-traumatic stress disorder following severe traumatic brain injury. *Brain Injury*.

[CIT0015] Bryant R. A., Marosszeky J. E., Crooks J., Gurka J. A. (2000). Posttraumatic stress disorder after severe traumatic brain injury. *American Journal of Psychiatry*.

[CIT0016] Bryant R. A., Moulds M. L., Guthrie R. M. (2000). Acute stress disorder scale: A self-report measure of acute stress disorder. *Psychological Assessment*.

[CIT0017] Chung M. C., Preveza E., Papandreou K., Prevezas N. (2007). Locus of control among spinal cord injury patients with different levels of posttraumatic stress disorder. *Psychiatry Research*.

[CIT0018] Copes W. S., Champion H. R., Sacco W. J., Lawnick M. M., Keast S. L., Bain L. W. (1988). The injury severity score revisited. *Journal of Trauma*.

[CIT0019] Creamer M., O'Donnell M. L., Pattison P. (2004). The relationship between acute stress disorder and posttraumatic stress disorder in severely injured trauma survivors. *Behaviour Research and Therapy*.

[CIT0020] Cuthbertson B. H., Hull A., Strachan M., Scott J. (2004). Post-traumatic stress disorder after critical illness requiring general intensive care. *Intensive Care Medicine*.

[CIT0021] Davydow D. S., Gifford J. M., Desaim S. V., Needham D. M., Bienvenu O. J. (2008). Posttraumatic stress disorder in general intensive care unit survivors: A systematic review. *General Hospital Psychiatry*.

[CIT0022] Davydow D. S., Zatzick D. F., Rivara F. P., Jurkovich G. J., Wang J., Roy-Byrne P. P., MacKenzie E. J. (2009). Predictors of posttraumatic stress disorder and return to usual major activity in traumatically injured intensive care unit survivors. *General Hospital Psychiatry*.

[CIT0023] Deja M., Denke C., Weber-Carstens S., Schröder J., Pille C. E., Hokema F., Kaisers U. (2006). Social support during intensive care unit stay might improve mental impairment and consequently health-related quality of life in survivors of severe acute respiratory distress syndrome. *Critical Care*.

[CIT0024] Dougall A. L., Ursano R. J., Posluszny D. M., Fullerton C. S., Baum A. (2001). Predictors of posttraumatic stress among victims of motor vehicle accidents. *Psychosomatic Medicine*.

[CIT0025] Ehring T., Ehlers A., Cleare A. J., Glucksman E. (2008). Do acute psychological and psychobiological responses to trauma predict subsequent symptom severities of PTSD and depression?. *Psychiatry Research*.

[CIT0026] Elklit A. (1996). Coping Styles Questionnaire: A contribution to the validation of a scale for measuring coping strategies. *Journal of Personality and Individual Differences*.

[CIT0028] Elklit A., Christiansen D. M. (2010). ASD and PTSD in rape victims. *Journal of Interpersonal Violence*.

[CIT0029] Elklit A., Pedersen S. S., Jind L. (2001). The crisis support scale: Psychometric qualities and further validation. *Journal of Personality and Individual Differences*.

[CIT0030] Ely E. W., Stephens R. K., Jackson J. C., Thomason J. W., Truman B., Gordon S., Bernard G. R. (2004). Current opinions regarding the importance, diagnosis, and management of delirium in the intensive care unit: A survey of 912 healthcare professionals. *Critical Care Medicine*.

[CIT0031] Fromberger U., Stieglitz R. D., Straub S., Nyberg E., Schlickewei W., Kuner E., Berger M. (1999). The concept of “sense of coherence” and the development of posttraumatic stress disorder in traffic accident victims. *Journal of Psychosomatic Research*.

[CIT0032] Fuglsang A. K., Moergeli H., Hepp-Beg S., Schnyder U. (2002). Who develops acute stress disorder after accidental injuries?. *Psychotherapy and Psychosomatics*.

[CIT0033] Gibbs M. S. (1989). Factors in the victim that mediate between disaster and psychopathology: A review. *Journal of Traumatic Stress*.

[CIT0034] Girard T. D., Shintani A. K., Jackson J. C., Gordon S. M., Pun B. T., Henderson M. S., Ely E. W. (2007). Risk factors for post-traumatic stress disorder symptoms following critical illness requiring mechanical ventilation: A prospective cohort study. *Critical Care*.

[CIT0037] Harvey A. G., Bryant R. A. (2002). Acute stress disorder: A synthesis and critique. *Psychological Bulletin*.

[CIT0038] Hauer D., Weis F., Krauseneck T., Vogeser M., Schelling G., Roozendaal B. (2009). Traumatic memories, post-traumatic stress disorder and serum cortisol levels in long-term survivors of the acute respiratory distress syndrome. *Brain Research*.

[CIT0038a] Hepp U., Moergeli H., Büchi S., Wittmann L., Schnyder U. (2005). Coping with serious accidental injury: A one year follow-up study. *Psychotherapy & Psychosomatics*.

[CIT0039] Hepp U., Moergeli H., Buchim S., Bruchhaus-Steinertm H., Kraemer B., Senskym T., Schnyder U. (2008). Post-traumatic stress disorder in serious accidental injury: 3-year follow-up study. *The British Journal of Psychiatry*.

[CIT0040] Holbrook T. L., Anderson J. P., Sieber W. J., Browner D., Hoyt D. B. (1999). Outcome after major trauma: 12-month and 18-month follow-up results from the Trauma Recovery Project. *Journal of Trauma*.

[CIT0041] Jones C., Griffiths R. D., Humphris G., Skirrow P. M. (2001). Memory, delusions, and the development of acute posttraumatic stress disorder-related symptoms after intensive care. *Critical Care Medicine*.

[CIT0042] Joseph S., Andrews B., Williams R., Yule W. (1992). Crisis support and psychiatric symptomatology in adult survivors of the Jupiter cruise ship disaster. *British Journal of Clinical Psychology*.

[CIT0043] Kapfhammer H. P., Rothenhäusler H. B., Krauseneck T., Stoll C., Schelling G. (2004). Posttraumatic stress disorder and health-related quality of life in long-term survivors of acute respiratory distress syndrome. *American Journal of Psychiatry*.

[CIT0045] King N. S. (2008). PTSD and traumatic brain injury: Folklore and fact?. *Brain Injury*.

[CIT0046] Kress J. P., Gehlbach B., Lacy M., Pliskin N., Pohlman A. S., Hall J. B. (2003). The long-term psychological effects of daily sedative interruption on critically ill patients. *American Journal of Respiratory and Critical Care Medicine*.

[CIT0047] Kushner M. G., Riggs D. S., Foa E. B., Miller S. M. (1992). Perceived controllability and the development of posttraumatic stress disorder (PTSD) in crime victims. *Behavior Research and Therapy*.

[CIT0049] Mollica R. F., Caspi-Yavin Y., Bollini P., Truang T., Tor S., Lavelle J. (1992). The Harvard Trauma Questionnaire: Validating a cross-cultural instrument for measuring torture, trauma, and posttraumatic stress disorder in Indochinese refugees. *The Journal of Nervous and Mental Disease*.

[CIT0051] Nielsen M. S. (2003). Crisis support and coping as mediators of well-being in persons with spinal cord lesion. *Journal of Clinical Psychology in Medical Settings*.

[CIT0052] Novaes M. A. F. P., Knobel E., Bork A. M., Pavão O. F., Nogueira-Martins L. A., Ferraz M. B. (1999). Stressors in ICU: Perception of the patient, relatives and health care team. *Intensive Care Medicine*.

[CIT0053] O'Donnell M. L., Creamer M., Bryant R. A., Schnyder U., Shalev A. (2003). Posttraumatic disorders following injury: An empirical and methodological review. *Clinical Psychology Review*.

[CIT0054] O'Donnell M. L., Creamer M., Holmes A. C., Ellen S., McFarlane A. C., Judson R., Bryant R. A. (2010). Posttraumatic stress disorder after injury: Does admission to intensive care unit increase risk?. *Journal of Trauma*.

[CIT0055] Ozer E. J., Best S. R., Lipsey T. L., Weiss D. S. (2003). Predictors of posttraumatic stress disorder and symptoms in adults: A meta-analysis. *Psychological Bulletin*.

[CIT0056] Rattray J. E., Johnston M., Wildsmith J. A. (2005). Predictors of emotional outcomes of intensive care. *Anaesthesia*.

[CIT0057] Ratzer M., Romano E., Elklit A. (2014). Posttraumatic stress disorder in patients following intensive care unit treatment: A review of studies regarding prevalence and risk factors. *Journal of Treatment and Trauma*.

[CIT0058] Rimel R. W., Giordani B., Barth J. T., Jane J. A. (1982). Moderate head injury: Completing the clinical spectrum of brain trauma. *Neurosurgery*.

[CIT0059] Roberts B. (2004). Screening for delirium in an adult intensive care unit. *Intensive and Critical Care Nursing*.

[CIT0060] Roberts B. L., Rickard C. M., Rajbhandari D., Reynolds P. (2006). Patients’ dreams in ICU: Recall at two years post discharge and comparison to delirium status during ICU admission: A multicentre cohort study. *Intensive and Critical Care Nursing*.

[CIT0061] Roger D., Jarvis G., Najarian B. (1993). Detachment and coping: The construction and validation of a new scale for measuring coping strategies. *Personality and Individual Differences*.

[CIT0062] Rotter J. B. (1966). Generalized expectancies for internal versus external control of reinforcement. *Psychological Monographs*.

[CIT0063] Sackey P. V., Martling C. R., Carlswärd C., Sundin O., Radell P. J. (2008). Short- and long-term follow-up of intensive care unit patients after sedation with isoflurane and midazolam-a pilot study. *Critical Care Medicine*.

[CIT0064] Samuelson K. A., Lundberg D., Fridlund B. (2007). Stressful memories and psychological distress in adult mechanically ventilated intensive care patients – a 2-month follow-up study. *Acta Anaesthesiolica Scandinavica*.

[CIT0065] Schelling G., Briegel J., Roozendaal B., Stoll C., Rothenhäusler H. B., Kapfhammer H. P. (2001). The effect of stress doses of hydrocortisone during septic shock on posttraumatic stress disorder in survivors. *Biological Psychiatry*.

[CIT0066] Schelling G., Kilger E., Roozendaal B., de Quervain D. J., Briegel J., Dagge A., Kapfhammer H. P. (2004). Stress doses of hydrocortisone, traumatic memories, and symptoms of posttraumatic stress disorder in patients after cardiac surgery: A randomized study. *Biological Psychiatry*.

[CIT0067] Schelling G., Stoll C., Kapfhammer H. P., Rothenhäusler H. B., Krauseneck T., Durst K., Briegel J. (1999). The effect of stress doses of hydrocortisone during septic shock on posttraumatic stress disorder and health-related quality of life in survivors. *Critical Care Medicine*.

[CIT0068] Schnyder U., Moergeli H., Klaghofer R., Buddeberg C. (2001). Incidence and prediction of posttraumatic stress disorder symptoms in severely injured accident victims. *American Journal of Psychiatry*.

[CIT0068a] Shalev A. Y., Peri T., Canetti L., Schreiber S. (1996). Predictors of PTSD in injured trauma survivors: A prospective study. *American Journal of Psychiatry*.

[CIT0069] Skalski C. A., DiGerolamo L., Gigliotti E. (2006). Stressors in five client populations: Neuman systems model-based literature review. *Journal of Advanced Nursing*.

[CIT0070] Sternbach G. L. (2000). The Glasgow coma scale. *The Journal of Emergency Medicine*.

[CIT0071] Stoll C., Schelling G., Goetz A. E., Kilger E., Bayer A., Kapfkammer H. P., Peter K. (2000). Health-related quality of life and post-traumatic stress disorder in patients after cardiac surgery and intensive care treatment. *The Journal of Thoracic and Cardiovascular Surgery*.

[CIT0072] Teasdale G., Jennett B. (1974). Assessment of coma and impaired consciousness. A practical scale. *Lancet*.

[CIT0074] Weinert C. R., Sprenkle M. (2008). Post-ICU consequences of patient wakefulness and sedative exposure during mechanical ventilation. *Intensive Care Medicine*.

[CIT0075] Weis F., Kilger E., Roozendaal B., de Quervain D. J., Lamm P., Schmidt M., Schelling G. (2006). Stress doses of hydrocortisone reduce chronic stress symptoms and improve health-related quality of life in high-risk patients after cardiac surgery: A randomized study. *Journal of Thoracic and Cardiovascular Surgery*.

[CIT0076] World Medical Association (2000).

[CIT0077] Zatzick D. F., Rivara F. P., Nathens A. B., Jurkovich G. J., Wang J., Fan M. Y., Mackenzie E. J. (2007). A nationwide US study of post-traumatic stress after hospitalization for physical injury. *Psychological Medicine*.

